# Probiotic-derived silver nanoparticles target mTOR/MMP-9/BCL-2/dependent AMPK activation for hepatic cancer treatment

**DOI:** 10.1007/s12032-024-02330-8

**Published:** 2024-04-04

**Authors:** Alaa Elmetwalli, Mohamed O. Abdel-Monem, Ali H. El-Far, Gehad S. Ghaith, Noaf Abdullah N. Albalawi, Jihan Hassan, Nadia F. Ismail, Tarek El-Sewedy, Mashael Mashal Alnamshan, Nouf K. ALaqeel, Ibtesam S. Al-Dhuayan, Mervat G. Hassan

**Affiliations:** 1 Department of Clinical Trial Research Unit and Drug Discovery, Egyptian Liver Research Institute and Hospital (ELRIAH), Mansoura, Egypt; 2 Microbiology Division, Higher Technological Institute of Applied Health Sciences, Egyptian Liver Research Institute and Hospital (ELRIAH), Mansoura, Egypt; 3 Botany and Microbiology Department, Faculty of Science, Benha University, Benha, Egypt; 4 Department of Biochemistry, Faculty of Veterinary Medicine, Damanhour University, Damanhour, 22511 Egypt; 5 Department of Nursing, Al Hadithah General Hospital, Al Qurayyat, Saudi Arabia; 6 Department of Applied Medical Chemistry, Medical Research Institute, Alexandria University, Alexandria, Egypt; 7 Health Information Management Program, Biochemistry, Faculty of Health Science Technology, Borg El Arab Technological University, Alexandria, Egypt; 8 Biology Department, College of Science, Imam Abdulrahman Bin Faisal University, 31441 Dammam, Saudi Arabia

**Keywords:** AgNPs, Apoptosis, Hepatic cancer, *Lactobacillus acidophilus*, α-SMA, HepG2

## Abstract

Recent advances in nanotechnology have offered novel ways to combat cancer. By utilizing the reducing capabilities of *Lactobacillus acidophilus*, silver nanoparticles (AgNPs) are synthesized. The anti-cancer properties of AgNPs have been demonstrated in previous studies against several cancer cell lines; it has been hypothesized that these compounds might inhibit AMPK/mTOR signalling and BCL-2 expression. Consequently, the current research used both in vitro and in silico approaches to study whether *Lactobacillus acidophilus* AgNPs could inhibit cell proliferation autophagy and promote apoptosis in HepG2 cells. The isolated strain was identified as *Lactobacillus acidophilus* strain RBIM based on 16 s rRNA gene analysis. Based on our research findings, it has been observed that this particular strain can generate increased quantities of AgNPs when subjected to optimal growing conditions. The presence of silanols, carboxylates, phosphonates, and siloxanes on the surface of AgNPs was confirmed using FTIR analysis. AgNPs were configured using UV–visible spectroscopy at 425 nm. In contrast, it was observed that apoptotic cells exhibited orange-coloured bodies due to cellular shrinkage and blebbing initiated by AgNP treatment, compared to non-apoptotic cells. It is worth mentioning that AgNPs exhibited remarkable selectivity in inducing cell death, specifically in HepG2 cells, unlike normal WI-38 cells. The half-maximum inhibitory concentration (IC_50_) values for HepG2 and WI-38 cells were 4.217 µg/ml and 154.1 µg/ml, respectively. AgNPs induce an upregulation in the synthesis of inflammation-associated cytokines, including (TNF-α and IL-33), within HepG2 cells. AgNPs co-treatment led to higher glutathione levels and activating pro-autophagic genes such as AMPK.

Additionally, it resulted in the suppression of mTOR, MMP-9, BCL-2, and α-SMA gene expression. The docking experiments suggest that the binding of AgNPs to the active site of the AMPK enzyme leads to inhibiting its activity. The inhibition of AMPK ultimately results in the suppression of the mechanistic mTOR and triggers apoptosis in HepG2 cells. In conclusion, the results of our study indicate that the utilization of AgNPs may represent a viable strategy for the eradication of liver cancerous cells through the activation of apoptosis and the enhancement of immune system reactions.

**Supplementary Information:**

The online version contains supplementary material available at 10.1007/s12032-024-02330-8.

## Introduction

Cancer is a global health issue affecting millions of people worldwide. Liver cancer, in particular, has become a significant concern due to its high mortality rate [1, 2]. Traditional cancer treatment methods often come with severe side effects, highlighting the need for alternative therapeutic approaches [3–5]. In recent years, nanotechnology has emerged as a promising field in cancer research, offering novel ways to combat this devastating disease [6]. The synthesis of silver nanoparticles (AgNPs) from *Lactobacillus acidophilus* involves a bio-reduction approach that utilizes the reducing properties of the bacterial cells [7].

In bacteria-synthesized AgNPs, the arrangement of Ag atoms varies significantly from species to species [8]. Those differences can be attributed to the fact that different microorganisms have different enzymes that are involved in the reduction of Ag-oxyanions. These enzymes can modify the structure, size, and shape of AgNPs and affect their properties [9]. Furthermore, the bacterial species can also influence the environment in which the AgNPs are produced, thus further influencing their characteristics [10]. Among these bacteria is *Lactobacillus acidophilus*, a probiotic bacterium that has gained significant attention for its potential anti-cancer properties [11]. Researchers have utilized *Lactobacillus acidophilus* to synthesize AgNPs due to its ability to reduce silver ions into their nanoparticle form [7, 12]. The synthesis process involves the use of *Lactobacillus acidophilus* culture filtrate, which acts as a reducing and stabilizing agent for the formation of AgNPs. The resulting nanoparticles exhibit unique physicochemical properties that make them suitable for various biomedical applications, including cancer therapy [13].

The pharmaceutical and biological potential of green-synthesized AgNPs has gained attention due to their unique properties [14]. The following are some of their effects; an array of viruses are susceptible to the antiviral agents in AgNPs, including influenza and herpes simplex virus. Their function is to inhibit viral attachment and entry into host cells. In addition, they inhibit viral protein synthesis, making them potentially effective against viruses [15]. A biofilm is a community of microorganisms encased in an extracellular polymer matrix that resists antibiotics. Biofilms are disrupted, microbial adhesion is inhibited, and embedded bacteria are killed by AgNPs. As a result, they can prevent biofilm-associated infections, such as those associated with medical devices [16].

Analgesic effects of AgNPs have been explored. They reduce oxidative stress and inhibit pro-inflammatory mediators, modulating inflammatory responses. In addition, AgNPs can enhance the effectiveness of conventional pain medications [17]. The efficacy and safety of these drugs must be validated in clinical studies. The anticoagulant properties of AgNPs are thought to arise from the interaction of AgNPs with blood components. Thrombus formation can be prevented by inhibiting platelet aggregation and adhesion to vascular surfaces. Additionally, AgNPs interfere with factors involved in blood clotting, modulating the coagulation cascade [18].

AgNPs possess unique properties due to their small size and large surface area-to-volume ratio. Their nanoscale dimensions allow for better penetration into cancer cells, enhancing their therapeutic potential [19]. The increased surface area enables efficient interaction with cellular components, aiding in their anti-cancer activity. AgNPs have been shown to be readily taken up by liver cancer cells. Once inside the cells, they can interact with various cellular targets, triggering apoptotic pathways. This cellular uptake is facilitated by the nanoparticles' small size and surface modifications, which can enhance their internalization [20]. AgNPs have been reported to induce mitochondrial dysfunction in liver cancer cells, further contributing to their apoptotic effects [21, 22]. These AgNPs can disrupt the mitochondrial membrane potential, impairing energy production and triggering apoptotic signalling pathways. This disruption of mitochondrial function is considered a crucial step in the induction of apoptosis [23].

Apoptosis, or programmed cell death, is a tightly regulated process responsible for maintaining tissue homeostasis. Dysregulation of apoptosis can contribute to cancer development and progression [24–26]. AgNPs derived from *Lactobacillus acidophilus* have been found to induce apoptotic cell death in liver cancer cells [27]. They activate various signalling pathways and promote the release of pro-apoptotic proteins, leading to the fragmentation and subsequent death of cancer cells. This apoptotic cell death induction offers a potential therapeutic strategy for liver cancer treatment [28, 29].

The AMP-activated protein kinase (AMPK) and mammalian target of rapamycin (mTOR) signalling pathway is a crucial cellular pathway that regulates various cellular processes, including autophagy [30, 31]. Under normal conditions, mTOR inhibits autophagy by phosphorylating and inactivating autophagy-related proteins [32]. However, in the presence of cellular stress, such as nutrient deprivation or oxidative stress, AMPK is activated, leading to the inhibition of mTOR and subsequent induction of autophagy [33]. Matrix metalloproteinase-9 (MMP-9) is an enzyme that plays a crucial role in extracellular matrix remodelling and cell migration [34]. In liver cancer cells, MMP-9 has been found to interact with B-cell lymphoma 2 (BCL-2) [35], an anti-apoptotic protein that prevents cell death. The interaction between MMP-9 and BCL-2 can promote cell survival and contribute to cancer progression [36].

AgNPs have been shown to activate the AMPK/mTOR signalling pathway, leading to autophagy induction in liver cancer cells. AgNPs exert their effect by modulating the phosphorylation status of AMPK and mTOR [37]. Studies have demonstrated that AgNPs can increase AMPK phosphorylation and inhibit mTOR signalling, resulting in the induction of autophagy [38, 39]. This activation of the AMPK/mTOR pathway by AgNPs promotes the degradation of damaged proteins and organelles, ultimately leading to cell death in liver cancer cells [40]. Furthermore, AgNPs have also been found to regulate the MMP-9-BCL-2 signalling pathway [41]. Studies have shown that AgNPs can inhibit MMP-9 activity, thereby reducing its interaction with BCL-2 [42, 43]. This disruption of the MMP-9-BCL-2 interaction leads to decreased cell survival and increased susceptibility to apoptosis, further contributing to the anti-cancer effect of AgNPs in liver cancer cells [44].

As part of this study, we synthesized biogenic AgNPs using *Lactobacillus acidophilus*. Furthermore, *Lactobacillus acidophilus*-enriched nano-Ag was evaluated for its efficacy in suppressing liver cancer cells as dietary supplements/nutraceuticals. AgNPs were also investigated for their ability to induce apoptosis and contribute to the immune response against liver cancer cells. To accomplish this, we analyzed pro-apoptotic markers and observed the influence of AgNPs in human liver cancer cells (HepG2). As a result of AgNPs activating the apoptotic machinery, liver cancer cells were destroyed.

## Materials and methods

### *Lactobacillus acidophilus* isolation, identification, and molecular confirmation

Since *Lactobacillus acidophilus* is a non-toxic, environmentally benign probiotic microbe, it was used in this study after being isolated from fermented milk products. Two mL of fermented milk were inoculated with MRS Broth (Thermo Fisher Scientific, USA) and allowed to access for 24 h at 37 °C, serving as an enrichment average. The enriched samples were then added to MRS agar and allowed to hatch for 48 h in an anaerobic environment. The colonies were cleansed by other subcultures that came after their original subculture. Through the use of primers encoding members of the facilitator family, which is a large family of membrane transport proteins (F: CATGTTGGGATGCAATGAG, R: TTTCAAAACTTGTCCTGCTG), the existence of *Lactobacillus acidophilus* was molecularly confirmed [7]. Illustratively, 2 µL of template DNA, 1 µL of each primer (25 µM), and 25 µL of 2 × Taq PCR Master Mix were used in the PCR reaction. An additional 50 µL of sterilized water was added. The DNA double helix melts at 95°C during the denaturing phase. Denaturing, annealing, and elongation cycles come after this step. Separation of the DNA strands occurs at 95°C during the denaturing stage. To enable primer binding to template DNA, the annealing step is performed at 55°C. The complementary strand of DNA may be synthesized by DNA polymerase at 72°C thanks to the elongation cycle. Last but not least, the reaction is terminated at 4°C after the previous extension step permits any leftover DNA strands to be finished at 72°C. The isolate's 16S rRNA gene sequences were compared to reference database sequences in MEGA version 5.0 using the BLAST method [45]. The accession number for the closest match was found at https://www.ncbi.nlm.nih.gov/nuccore/OR616671.1. As a result, the isolates could be placed in the proper taxonomic position while building the phylogenetic tree.

### The extraction process of AgNPs

To isolate AgNPs from bacteria, we adapted the method proposed by Keskin et al. [46]. First, *Lactobacillus acidophilus*-derived nano-Ag bacteria were harvested from MRS broth by centrifugation (1700×*g*, 15 min, 4ºC). As a result of three PBS washes and 90 min of incubation at 37ºC, bacteria were resuspended in NaOH 0.1 M (5 ml of NaOH was used per 0.1 g of bacteria). After several washes with de-ionized water (6000×*g*, 15 min, 4ºC), the lysed bacteria and AgNPs-clusters were recovered. Sonication was performed on harvested clusters containing AgNPs using the UP50H sonifier from Hielscher Ultrasonics GmbH (Teltow, Germany). Finally, vacuum filtration using membrane filters with pore sizes ranging from 2 to 0.45 mm was used to separate the dispersed AgNPs from cell debris. AgNPs were stored at 4ºC as a purified aqueous solution.

### Characterization of AgNPs

#### Fourier transform infrared spectroscopy (FTIR)

Under the Japanese Alpha e-Bruker spectrometer, AgNPs were investigated at a resolution of 4 cm^−1^ and a frequency of 1000–4000 wave numbers cm^–1^. Following the acquisition of smoothed spectra, baselines were modified [47]. Comparison with publicly accessible standards was used to analyze the FTIR spectra.

### UV–Vis spectroscopy of AgNPs

Using a multi-plate reader from Perkin Elmer, we measured the absorption spectra of purified AgNPs. 96-well plates were seeded with 100 ml of AgNPs suspended in de-ionized water per well. Normalized readings were taken against de-ionized water-filled blank wells [48].

### Crystallography and transmission electron microscopy (TEM)

XRD diffractograms of AgNPs were obtained using Cu-Kα radiation (*λ* = 1.5405 Å), and they were analyzed based on the Rietveld method in a Bragg–Brentano geometry, and the structure of the composites was characterized using the XRD diffractograms [49]. Using TEM (JEOL 2010, Tokyo, Japan), the AgNPs were examined as per the recommended procedure [50]. A carbon-coated copper grid was used in the TEM for mounting PG-AgNPs. Once the sample had been air-dried at room temperature, its size and shape were assessed at an acceleration voltage of 200kV.

### Determination of apoptosis

The HepG2 cells that had been given the AgNPs treatment were collected and given a pH 7.2, 1 × PBS wash [51]. Following that, cells were stained on clean microscope coverslips with acridine orange (AO) (50 µL of 1 mg/ml) and ethidium bromide (EB) (100 µL of 1 mg/ml). Cells were gathered and seen using a fluorescence microscope (ZOE fluorescent cell imager) after incubation for 20 min at 37º C in the dark (BioRad, USA).

### M30 apoptosense assay

The M30 Apoptosense® was used to assess apoptosis levels in HepG2 cells, following the established technique [52]. The quantification of soluble apoptosis-related and caspase-cleaved K18 fragments, including the neo-epitope K18Asp396 in soluble coexpression systems, may be achieved by the use of the M30 Apoptosense test. The technique used is a solid-phase sandwich enzyme immunoassay, in which the samples undergo a reaction with an anti-K18 solid-phase capture antibody. This is followed by the interaction with the HRP (horseradish peroxidase) conjugated M30 antibody, specifically designed to target the K18Asp396 neo-epitope. The relationship between the absorbance measured at a wavelength of 450 nm using a Spectrophotometer and the concentration of the analyte is directly proportional.

### Cell culture and treatment

The culture of HepG2 and WI-38 human lung cells involved the use of DMEM medium (Lonza, Bio Whittaker®, USA) (CAS-No. 12614), which was supplemented with 10% fetal bovine serum (FBS) (Sigma, USA) (CAS-No. 1943609-65-1). Additionally, the medium was supplemented with 100 mg/ml penicillin and 100 mg/ml streptomycin and maintained at a temperature of 37 °C in a humidified incubator with 5% CO_2_. The treatments were consistently administered to cells that were 40–50% confluent after the subculturing of the cells at an 80%-90% confluency and subsequent splitting at a ratio of 1:6. All treatments were administered for a duration of 48 h, with drug dosages equivalent to half of the 50% inhibitory concentration (IC_50_) values (IC_50_/2). To conduct statistical analysis, three separate cell culture experiments were carried out, with each experiment being replicated three times [2, 25].

### Cell viability assay

In this study, the culture of HepG2 and WI-38 human lung cells involved the utilization of DMEM medium (Lonza, Bio Whittaker®, USA) (CAS-No. 12614). This medium was supplemented with 10% fetal bovine serum (FBS) (Sigma, USA) (CAS-No. 1943609-65-1), as well as 100 mg/ml penicillin and 100 mg/ml streptomycin. The cells were maintained in a humidified incubator at 37 °C, with the addition of 5% CO_2_ to the medium. The treatments were consistently administered to cells that had reached a confluence level of 40–50%. Prior to treatment, the cells were subcultured when they got a confluence level of 80%–90% and were divided at a ratio of 1:6.

The vitality of HepG2 and WI-38 cells was assessed using an MTT colourimetric test kit obtained from Sigma-Aldrich, USA. The effects of AgNPs and Sor on cell viability were investigated. The cells were grown overnight in 96-well plates at a density of 5 × 103 cells per well after seeding. Subsequently, the cells were treated with varying concentrations of AgNPs and Sor (ranging from 0.5 to 100 ng/ml) for a duration of 48 h. A volume of 100 ml of MTT working solution was added to each well, and the plates were incubated for a period of four hours at a temperature of 37°C in the absence of light. Following the removal of the medium, DMSO was introduced into each well and allowed to incubate for a duration of five minutes, facilitating the dissolution of the purple formazan crystals. The optical density (OD) at a wavelength of 570 nm was measured using a BIO-RAD PR4100 microplate reader manufactured in the United States. To determine the IC_50_, the vitality of cells subjected to treatment was compared to that of control cells that were not treated [26].

### Evaluation of the antioxidant capacity for AgNPs markers

The glutathione (GSH) levels in HepG2 and WI-38 cell lysates were evaluated after a 48-h treatment with half of the IC50 doses of AgNPs and Sor. The quantification of malondialdehyde (MDA) was conducted in cell lysates of both HepG2 and WI-38 cells to assess lipid peroxidation levels [25].

### Quantification of cytokines in AgNPs-treated cells supernatants

In HepG2 culture supernatants treated with AgNPs, the concentrations of IL-33 and TNF-α were assessed. 10,000 cells for HepG2 were planted in 96-well plates. After 24 h of adhesion, the cells were treated with a 50 µg/ml probiotic sample for 3 h [53]. ELISA was used to measure the number of cytokines in the culture supernatants. Results were revealed as the mean value ± standard deviation (SD) from at least three replicate tests. The cytokine levels in the culture supernatants were measured using the sandwich enzyme-linked immunosorbent assay. According to the manufacturer's instructions, a unique ELISA kit (bioscience, San Diego, CA, USA) for each cytokine was utilized. In an ELISA plate reader, the optical density of the reaction products was evaluated (EnSpire Multimode Plate Reader).

### RT-PCR assessment and RNA extraction

In accordance with the guidelines provided by the manufacturer, the extraction of total RNA from cells treated with a concentration equivalent to half of the IC50 was performed using the Gene JET RNA extraction kit (Cat#A0156) manufactured by Thermo Fisher Scientific. The purity and concentration of the extracted RNA were assessed using the Analytik Jena Scandrop200 instrument from Germany. Subsequently, cDNA synthesis was carried out using the SensiFASTTM cDNA Synthesis Kit (BIO-6505) from Thermo Co, USA.

Primers were developed for the AMPK, mTOR, MMP-9, BCL-2, and α-SMA genes in the context of quantitative RT-PCR. The primer sequences may be found in Supplementary Table S1. For the PCR reaction, a solution was prepared by combining 10µL of SYBR green, 2µL of cDNA template, and 6.4µL of nuclease-free water. The Q5plex detection device was used to detect rotor gene amplification. After an initial activation step at a temperature of 95°C for a duration of 2 min, a series of 45 cycles was conducted. Each cycle consisted of three steps: a denaturation step at 95°C for 5 s, an annealing step at 62°C for 10 s, and an extension step at 72°C for 20 s. The study used the comparative 2^−ΔΔC*t*^ technique to determine relative expression levels, with the internal housekeeping gene GAPDH serving as a reference [54].

### Molecular docking and ADMET

To determine the molecular interactions and scores of AgNO3-NPs and sorafenib against human MMP-9 and mTOR, the three-dimensional structures of MMP-9 and mTOR were retrieved from the RCSB Protein Data Bank (https://www.rcsb.org/) database. In addition, the AgNO3 and sorafenib structures were retrieved from PubChem (https://pubchem.ncbi.nlm.nih.gov/) database. Furthermore, the target proteins were prepared by removing water molecules and attaching ligands using the UCSF Chimera software package. The molecular docking interaction of AgNO3 and sorafenib against MMP-9 and mTOR was done using the AutoDock 4.2 tool and UCSF Chimera while visualized by BIOVIA Discovery Studio software. ADMET predictions of AgNO3 and sorafenib were made using the ADMET and toxicity tools present in BIOVIA Discovery Studio software [26].

### Analyses of statistics

Analysis of the data were performed with GraphPad Prism version 9.0 (GraphPad Software, Inc., La Jolla, CA, USA). An ANOVA was used to compare data between multiple groups, followed by Tukey's post-*hoc* test to compare data between individual groups. The least significance difference test was used to determine differences between means in all tests, with significance defined as *P* < 0.05 for all tests.

## Results

### Isolation, 16S rRNA gene sequence analysis, and biosynthesis of AgNPs

**Fig. 1:**
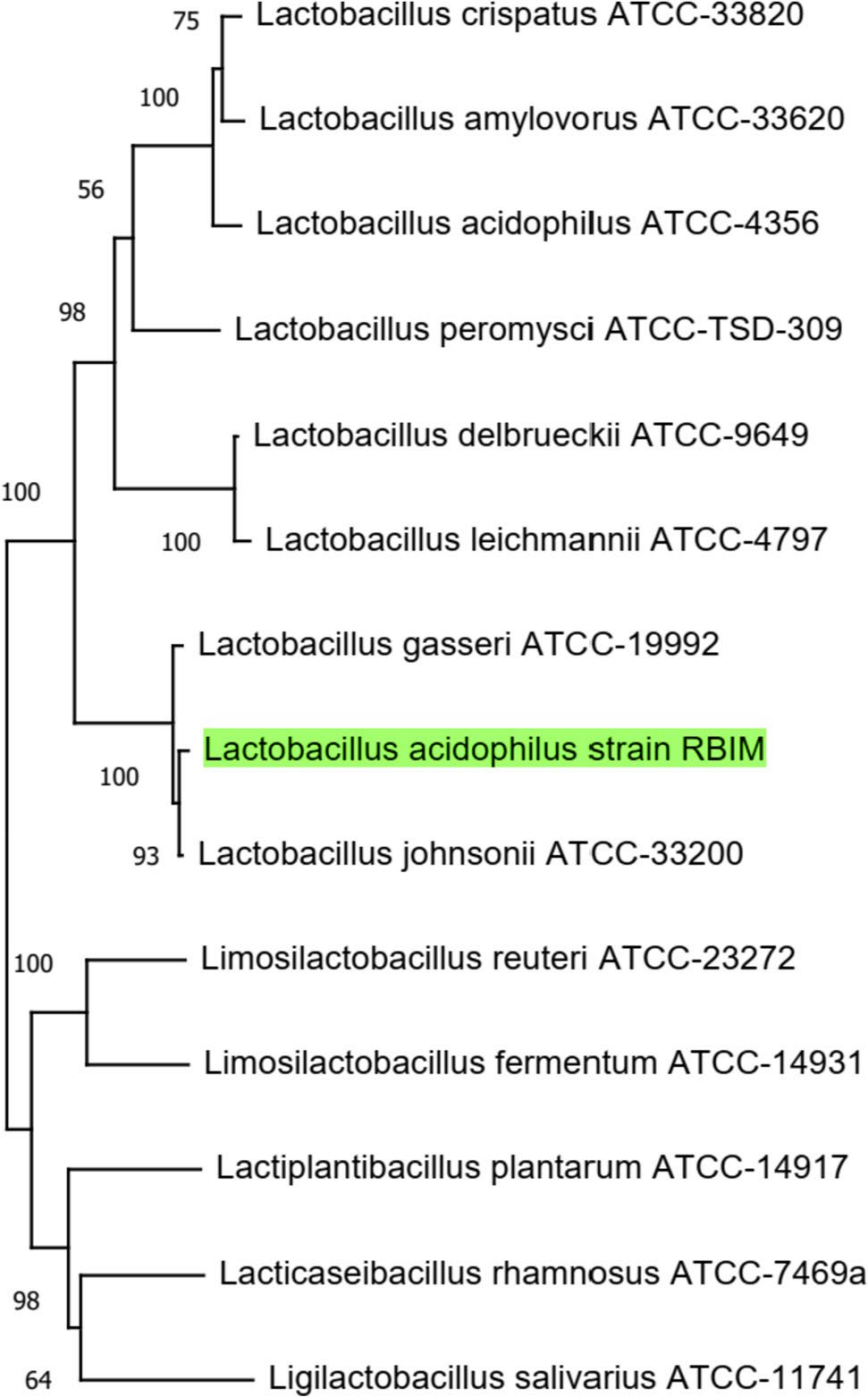
Analysis of the 16S rDNA of *Lactobacillus acidophilus* strain RBIM phylogenetic tree available in GenBank. The tree suggests that the strain RBIM belongs to the same species as other *Lactobacillus acidophilus* strains. Furthermore, the phylogenetic tree indicates that the strain RBIM is distinct from other *Lactobacillus* species

To verify identification through molecular analysis, the strain’s 16S rDNA gene was sequenced. The *Lactobacillus acidophilus* strain RBIM that has the highest similarity score to the unknown strain can be identified by applying the BLAST algorithm to compare a partial fragment of the strain’s 16S rDNA to a database of known 16S rDNA sequences. This was further supported by a phylogenetic tree constructed using the neighbour-joining method, which revealed that the unknown strain shared 99% similarities with the *Lactobacillus gasseri* ATCC-19992, allowing for identification as *Lactobacillus acidophilus* strain RBIM (Fig. 1). The sequence has been deposited in GenBank with accession number #OR616671.

### Characterization of AgNPs

#### Fourier-transform infrared spectroscopy (FTIR)

**Fig. 2:**
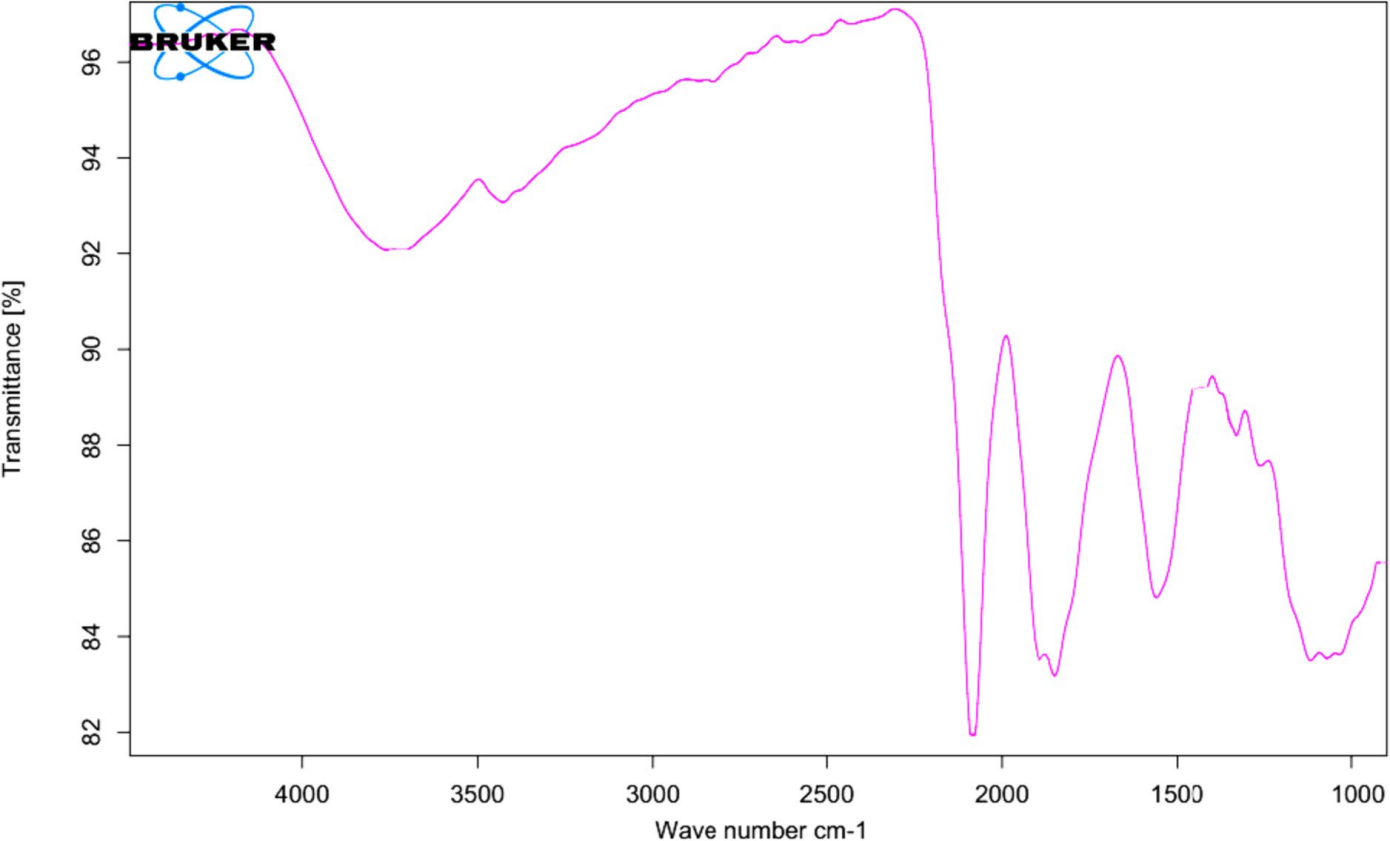
An FTIR spectrum of *Lactobacillus acidophilus* strain RBIM displays peaks at different wave numbers. *Lactobacillus acidophilus* strain RBIM exhibited various bands characteristic of AgNPs, which indicates that the AgNPs were highly effective at this specific frequency

FTIR measurements of dried AgNPs were used to evaluate how biomolecules interact with AgNPs, which may be responsible for their stability (capping material) in the medium. During the extraction process, biomolecules can consist of peptides, proteins, and carbohydrates. A well-known signature of the infrared region of the electromagnetic spectrum can be observed in proteins that have amide linkages between amino acid residues. An FTIR analysis was conducted to identify possible interactions between silver salts and protein molecules responsible for the reduction of Ag + ions and stabilization of AgNPs. The FTIR analysis of AgNPs was carried out between the wave numbers 1000 cm^ −1^ to 4000 cm^ −1^. The strong peaks at 3750 cm^ −1^, 3300 cm^ −1^, 2800 cm^ −1^, 2600 cm^ −1^, 1650 cm^ −1^, 1450 cm^ −1^, 1290 cm^ −1^, and 1070 cm^ −1^ were observed in the FTIR spectrum of AgNPs synthesized extracellularly by *Lactobacillus acidophilus* as shown in (Fig. 2). These peaks were attributed to the presence of silanols, carboxylates, phosphonates, and siloxanes on the surface of the AgNPs. The presence of these compounds was an indication of the biogenic synthesis of the silver nanoparticles.

Illustratively, the peaks observed at wave number 1650 cm^ −1^ are indicative of peptide linkage stretching. In contrast, 1290 cm^ −1^ demonstrated a stretching of CN-amines and a bending of the NH side of peptides. Peaks in this region can be attributed to the secondary structure of proteins, hydrogen bonding between amino acids, and electrostatic interactions between amino acids. Moreover, these peaks can give information about a protein’s flexibility, reactivity, and stability. The peak at 1070 cm^ −1^ demonstrates that –CH_3_ structurally bends in amino acids when the chain is stretched. It is likely that the peaks at 1450 ^cm–1^ are due to changes in the asymmetric stretch of NO caused by the presence of nitro compounds. This suggests that the nitro compounds may be affecting the overall structure of the amino acid, causing the –CH3 to structurally bend. This could explain why nitro compounds are responsible for the absorption spectrum shift. Furthermore, there are two peaks at 2600 cm^–1^ and 2800 cm^–1^, which are indicative of CH stretching in aldehydes. Due to the fact that saturated hydrocarbons have a –CH saturation vibration, we observed peaks at 3300 cm^–1^ and 3750 cm^–1^. This suggests that the aldehydes were more complex than the saturated hydrocarbons since they had additional CH stretching vibrations. The additional CH stretching vibrations in the aldehydes are necessary to accommodate the double bond, which requires more energy than the single bond in saturated hydrocarbons. This is why the nitro compounds are responsible for the shift in absorption spectra and why the CH stretching vibrations for the aldehydes are more complex than those in saturated hydrocarbons.

### UV–Vis spectroscopy of AgNPs

**Fig. 3:**
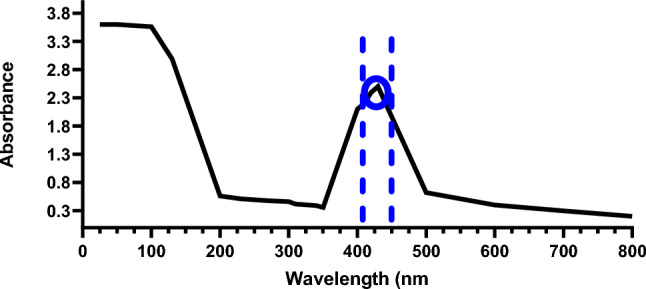
Analysis of AgNPs extractions by UV–Vis from lysed *Lactobacillus acidophilus* strain RBIM cultured in AgNO3 and dispersed in deionized water. The peak is located at 575–585 nm. In this peak, AgNPs were extracted from the solution, indicating that the synthesized AgNPs were successfully extracted

As part of a study on the derivation and stability of silver oxide nanoparticles in aqueous colloidal solutions, UV–visible spectral analysis was used to study their synthesis and stability. As can be seen from (Fig. 3), a strong peak has been observed at 425 nm. This peak was indicative of the formation and stability of silver oxide nanoparticles, providing further insight into the study. In general, the intensity increase could be interpreted as a result of a greater number of nanoparticles that are formed as a consequence of reducing silver nitrate that is present in aqueous solutions as a result of the reduction process.

### XRD and TEM analysis

**Fig. 4:**
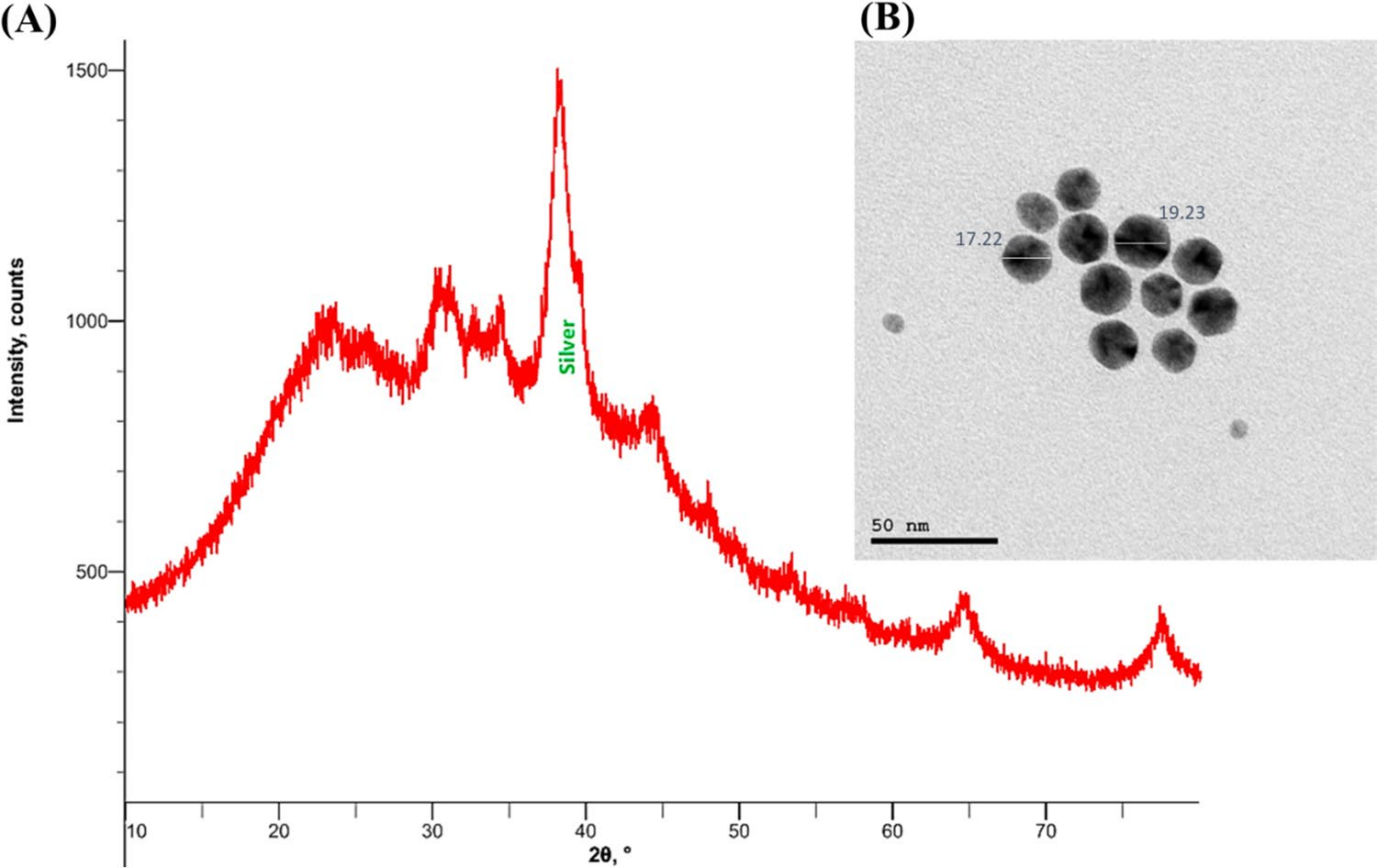
**A** AgNPs XRD spectrum recorded for *Lactobacillus acidophilus*. The spectrum presented peaks at 2θ values of 38.2°. These peaks are consistent with the crystalline structure of the AgNPs. This indicates that the AgNPs were successfully synthesized and interacted with the bacteria. **B** AgNPs micrographed by TEM. Approximately 17 nm in diameter with a standard deviation of 2 nm were the mean sizes of the AgNPs. Throughout the sample, the particles were uniformly distributed and spherical. TEM micrographs revealed that AgNPs were uniformly sized and distributed

The phase formation and purity of the nanoparticles could be ascertained by utilizing XRD measurement. Based on the XRD analysis (Fig. 4a**)**, it can be inferred that AgNPs are crystalline due to their narrow and sharp peaks**.** Furthermore, the XRD analysis also indicates that AgNPs are of high purity, as the peaks maintain their sharpness and intensity throughout the spectrum. A face-centred cubic structure is present in the silver nanocrystal, as indicated by intense peaks observed at 38° in the 2θ range. The intensity of the peaks also indicates that the particles are small and uniform in size, with a narrow size distribution. The shape of the peaks is also indicative of the crystallinity of the particles, as sharp peaks indicate that the particles are crystalline and uniform in size and shape**.** This corresponds to the silver file number 04-0783 on the Joint Committee on Powder Diffraction Standards (JCPDS) standard powder diffraction card. The TEM micrographs revealed a homogeneous size distribution of the AgNPs, suggesting that the particles were not aggregated and could retain their original shape (Fig. 4b). The particles' estimated sizes ranged from 17 to 19 nm, which is within the typical range for AgNPs. Additionally, the spherical form and good dispersion of AgNPs were revealed by the TEM micrograph. This indicates that the AgNPs are stable and appropriate for usage in a variety of applications as they are uniformly dispersed and of the right size and shape.

### Effect of AgNPs on apoptosis

**Fig. 5:**
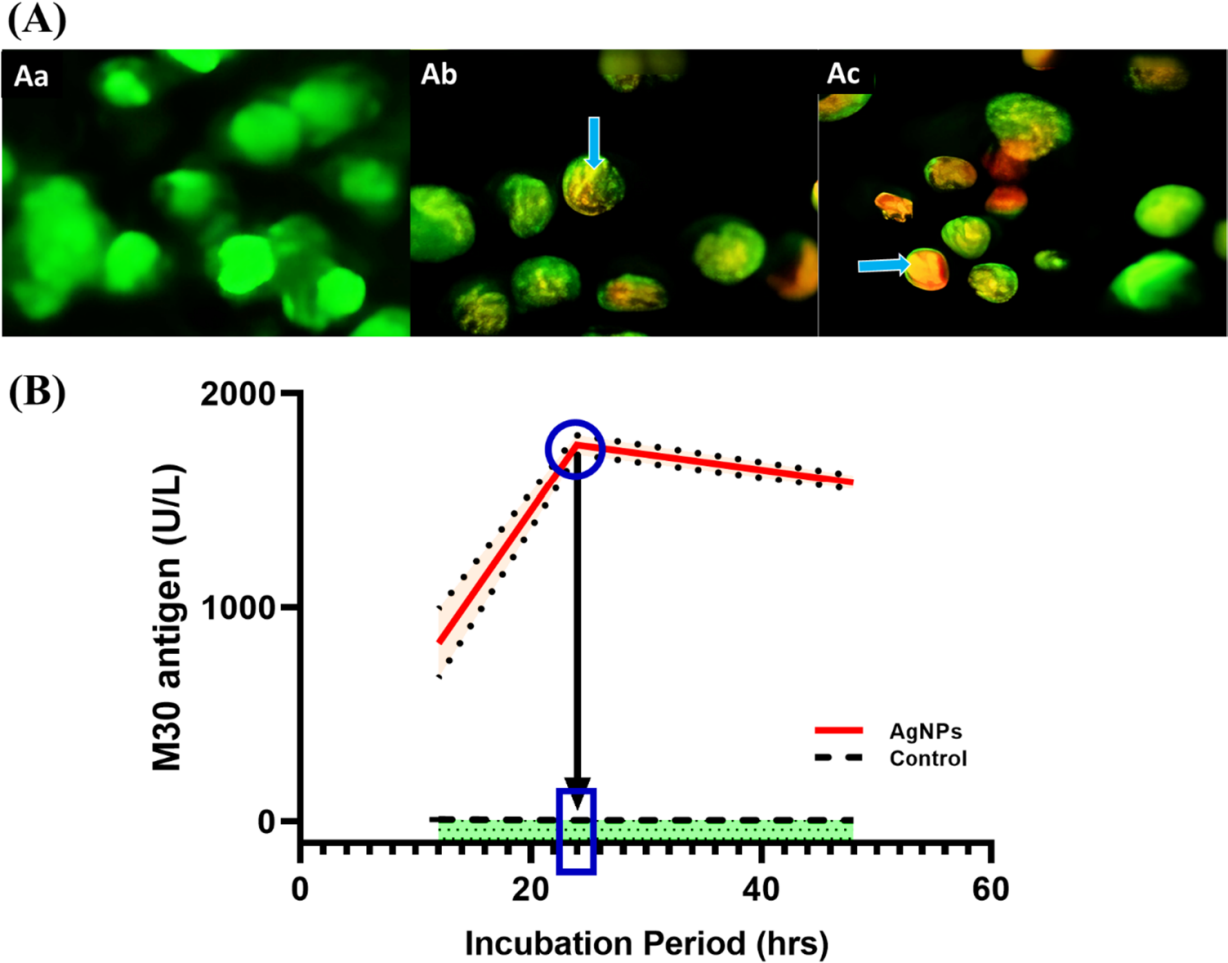
A AO/EB fluorescence images for (Ab) Control and (Ab),(AC) 50 μg/ml AgNPs-treated-HepG-2 cells. The arrows indicate that the cells are experiencing apoptosis, which results in chromatin condensation and fragmentation. Early-stage apoptosis is characterized by chromatin condensation, whereas late-stage apoptosis is characterised by chromatin fragmentation. **B** The amount of M30 in cell extracts. HepG2 cells were given an AgNPs treatment of 50 µg/ml over 24–48 h. The M30 epitope is also detected in HepG2 cells, indicating cell death. A higher level of these molecules was found in AgNPs-treated cells compared to controls, reaching a peak after 24 h. The graphs reveal the results as the mean ± SD of three replicates

In the treatment with AgNPs, the cells went through an apoptosis process, which was visualized by staining the cells with AO/EB. Observations of the fluorescence of normal viable control cells at the microscopic level revealed that the color of the cells was green due to the diffusion of an AO compound into the cell membrane. Apoptotic cells were, on the other hand, observed to have orange-coloured bodies due to the shrinkage and blebbing of the cells caused by the AgNP treatment compared to non-apoptotic cells. Apoptotic cells are typically characterized by yellow-green deposits of AO staining on the nuclei of early-stage apoptotic cells. As the apoptotic stage progressed, EB staining on the nuclei of late-stage apoptotic cells became more concentrated and asymmetrically localized. In necrotizing cells, the volume of the cells increased, and the fluorescence at the periphery of the cells was uneven and orange-red. There has been a suggestion that apoptosis is caused by AgNPs that are involved in disrupting the mitochondrial respiratory chain, resulting in reactive oxygen species (ROS) being produced and damaging nucleic acids. The uneven localization of EB staining in apoptotic cells suggests that the mitochondria are undergoing rapid and abnormal fragmentation, leading to the release of EB staining into the cytosol. In necrotizing cells, the ROS produced by the AgNPs further damages the mitochondria, leading to further fragmentation and the release of EB staining from the cells (Fig. 5a).

#### Induction of apoptosis in HepG2 treated with AgNPs

The concentrations of the M30 epitope in HepG2 cell extracts (Fig. 5b), which serve as an indicator of caspases 3/7/9 activation and subsequently apoptosis, were seen to be elevated in cells treated with AgNPs in comparison to the control group. The highest levels were observed after 23 h of treatment. This indicates that AgNPs can trigger apoptosis in HepG2 cells, potentially due to the oxidative stress caused by the AgNPs. The oxidative stress can activate the caspases, which in turn triggers the M30 epitope production and apoptosis. This suggests that AgNPs may be an effective therapeutic agent for treating certain cancers, as they can lead to apoptosis of the cancer cells. Additionally, AgNPs can be used in combination with other therapies, such as chemotherapy, to further increase their efficacy.

### Cytotoxicity evaluation

**Fig. 6:**
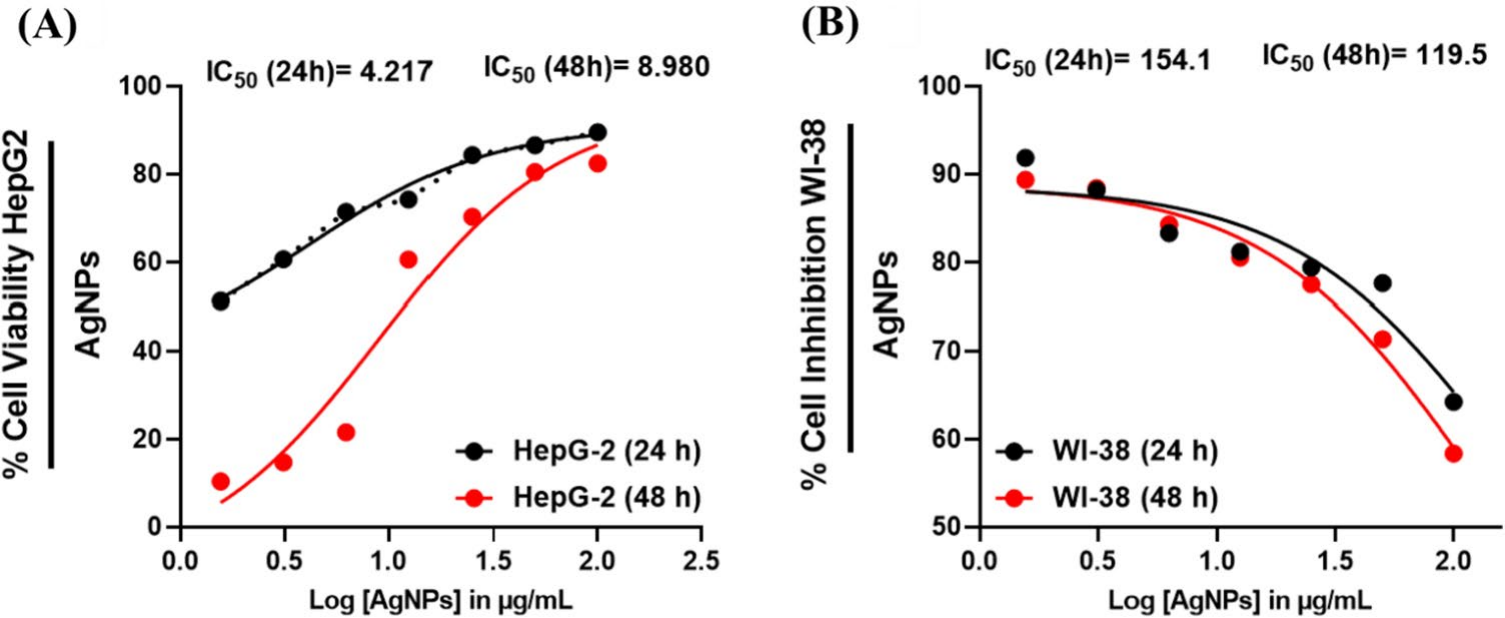
Growth inhibition curves in **A** HepG2 cells treated with AgNPs; **B** WI-38 cells treated with AgNPs. The MTT assay confirmed that the AgNPs generated a dose-dependent inhibition in cell proliferation in both cell lines, with more significant inhibitory activity in the HepG2 cells. Moreover, the inhibition was also prominent in HepG2 cells after 48 h of treatment. This implies that AgNPs may have a long-lasting effect on HepG2 cells

MTT tests were carried out against HepG2 and WI-38 cells to evaluate AgNPs’ cytotoxic potential. A range of 1.56–100 µg/ml of AgNPs was used to treat cells for 48 h. Both cell lines showed dose-dependent reductions in cell viability after 24 h of exposure to AgNPs. Assays with HepG2 and WI-38 demonstrated that AgNPs inhibited cell growth (IC50 4.217 µg/ml and 154.1 µg/ml, respectively) (Fig. 6). HepG2 and WI-38 cells, however, revealed reduced proliferation after 48 h post-AgNPs treatment (IC50 = 119.5 µg/ml and 8.98 µg/ml, respectively). This implies that AgNPs were more effective in reducing the growth of HepG2 cells than WI-38 cells after 48 h. This could be due to the fact that HepG2 cells are more susceptible than WI-38 cells to the effects of AgNPs. Additionally, the decrease in cell viability could be caused by the AgNPs binding to the cell membrane and interfering with the cell cycle.

### AgNPs treatment reduced MDA in the HepG2 cell line

**Fig. 7:**
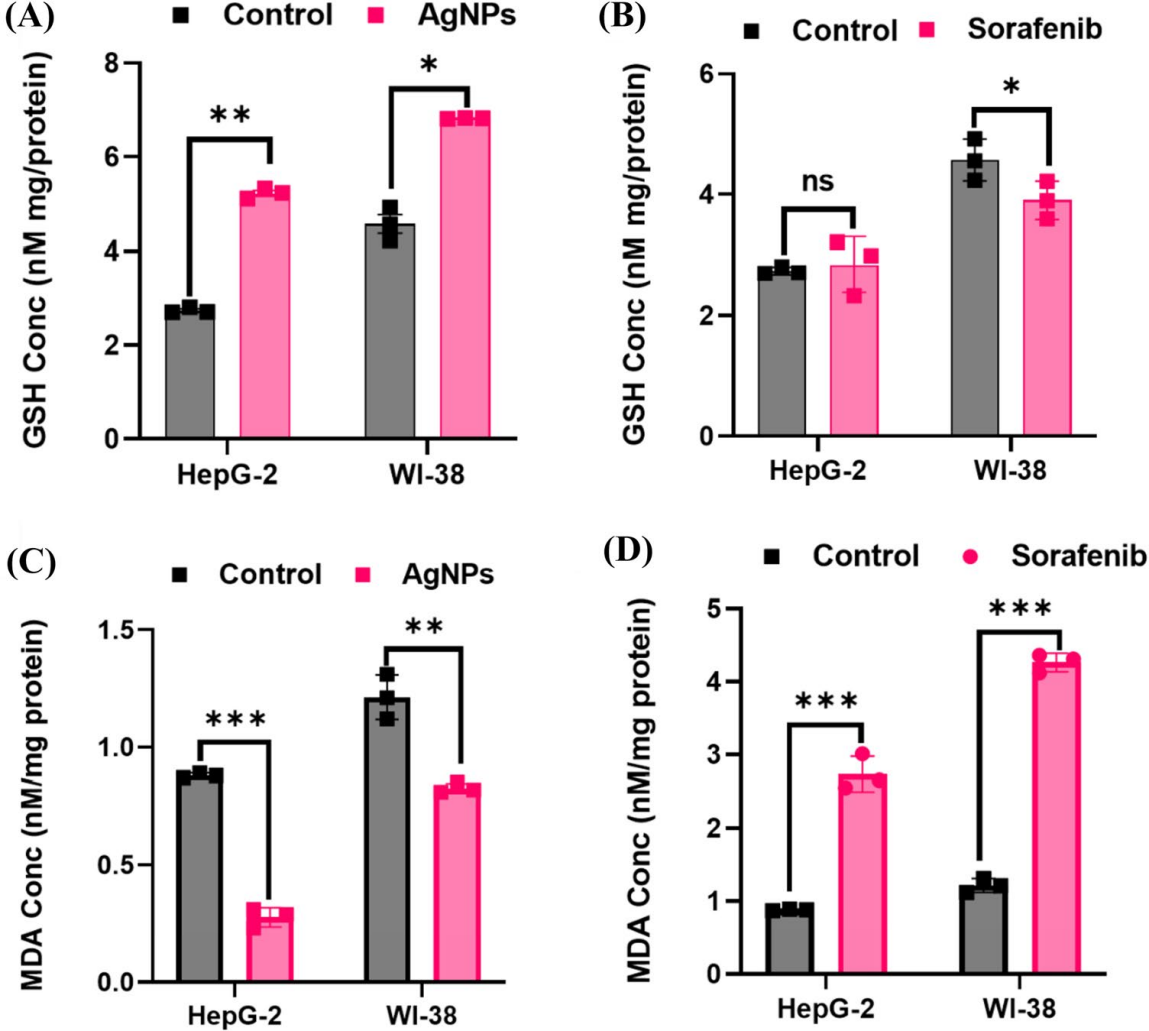
Oxidative stress markers and antioxidant activity of AgNPs and Sor **A** GSH of AgNPs in both HepG2 and WI-38 cell lines; **B** GSH of Sor in both HepG2 and WI-38 cell lines; **C** MDA concentration in control and treated HepG2 and WI-38 cells with AgNPs; **D** MDA concentration in control and treated HepG2 and WI-38 cells with Sor. *(*P* ≤ 0.05), **(*P* ≤ 0.01) and ***(*P* ≤ 0.001) means significantly different from control. The data were analyzed in triplicate and expressed as mean ± SD

In the HepG2 and WI-38 cell lines, it was shown that cells treated with AgNPs exhibited elevated levels of GSH compared to both the untreated control cells and the cells treated with Sor. As a result, the AgNPs therapy contributed to the most significant reduction of MDA levels in HepG2 cells as well as WI-38 cells when compared to a control solution. However, the MDA levels of HepG2 and WI-38 cells that were treated with Sor were significantly higher than those of the control cells (Fig. 7). This suggests that AgNPs may be a more effective therapy than Sor for reducing the MDA levels of HepG2 and WI-38 cells. Additionally, the GSH levels in the AgNPs-treated cells may have been increased to provide a defence against oxidative damage induced by the MDA.

### Influence of AgNPs-treated HepG2 Cells on the secretion of pro-inflammatory cytokines

**Fig. 8:**
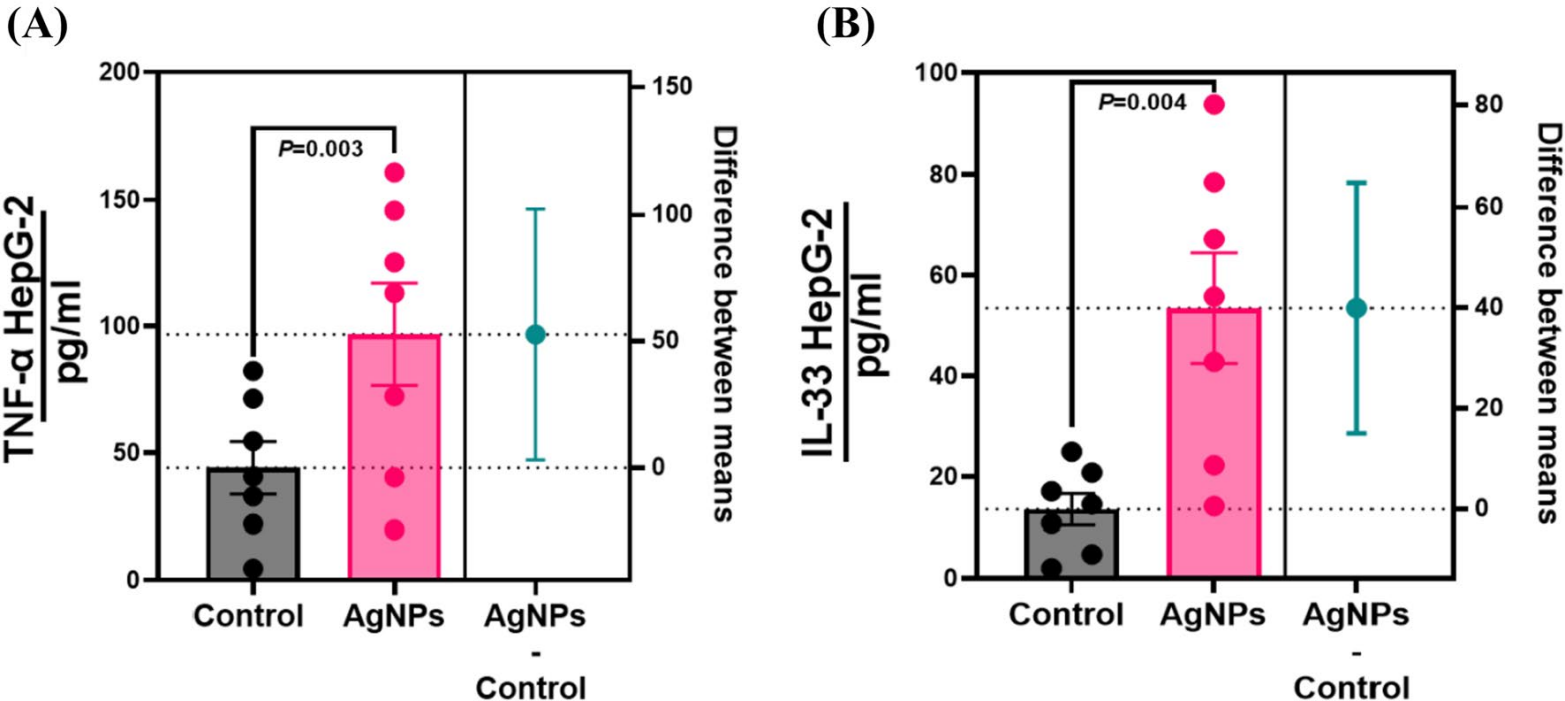
AgNPs induce pro-inflammatory cytokines in liver cancer cells. Following treatment with AgNPs, Liver cancer cells produce more cytokines that promote inflammation. **A** The production of TNF-α by AgNPs-treated HepG2 cells; **B** The production of IL-33 by AgNPs-treated HepG2 cells. AgNPs concentration of 50 µg/mL was added to the cells for 3 h. ELISA was used to determine the cytokine concentrations in the culture supernatants. The findings are the mean ± SD (*P* < 0.05, Student’s *t*-test) of three replicates

AgNPs cause an increase in the production of inflammation-related cytokines, such as TNF-α and IL-33, in HepG2 cells (Fig. 8). Immune response is enhanced by these cytokines, which are part of the body's natural response to infections and injuries. This stimulation is mediated by the activation of the AMPK pathway. The AMPK pathway is a crucial regulator of inflammation, and its activation leads to inflammatory cytokines. Therefore, the AMPK pathway is an important signaling component in the response to AgNPs.

### Effect of AgNPs on signalling pathways and apoptosis-autophagy-related marker genes

**Fig. 9:**
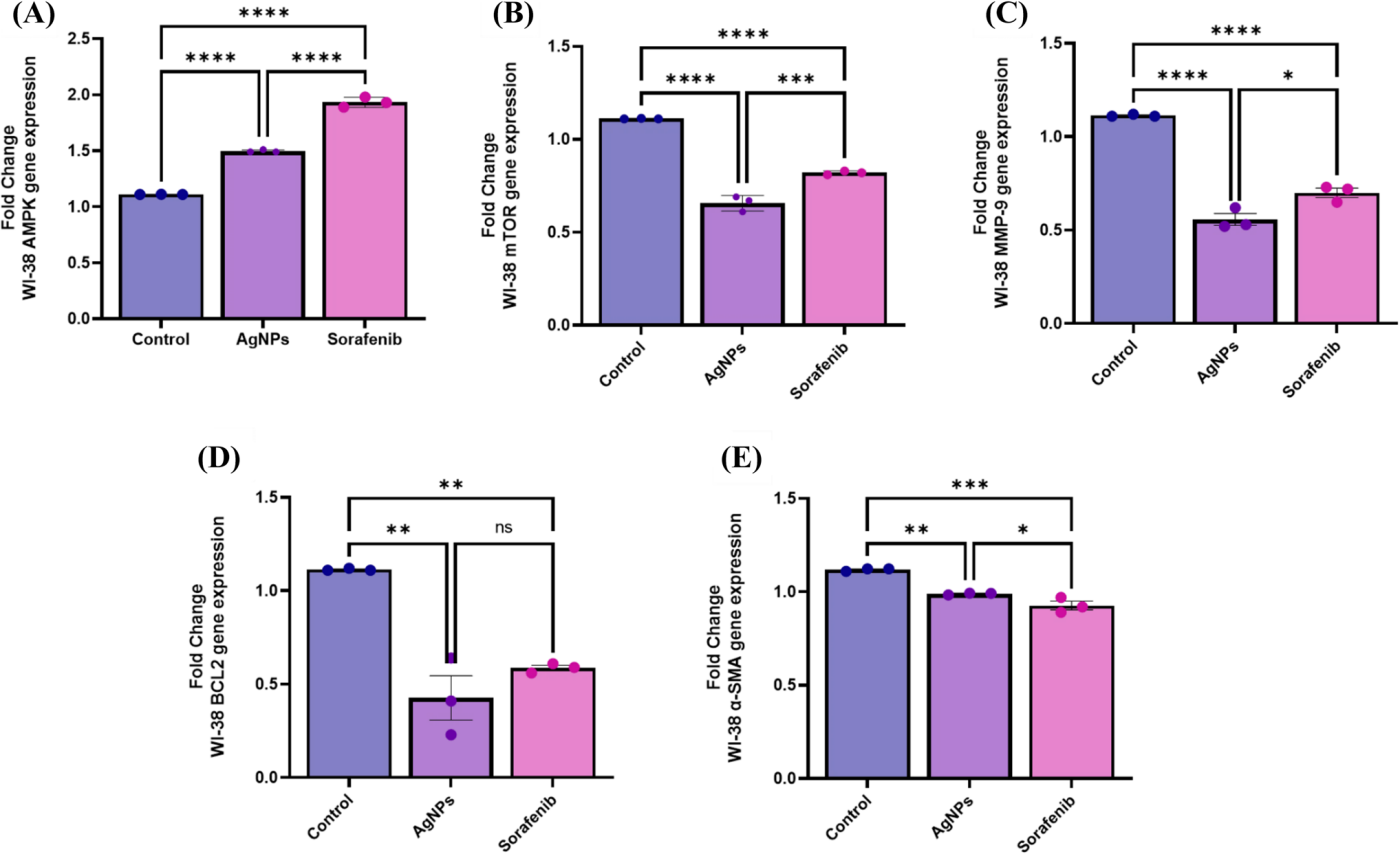
Effect of AgNPs or Sor treatment on the relative expression of apoptosis-autophagy-related genes on WI-38 normal cell lines **A** AMPK gene-fold change; **B** mTOR gene-fold change; **C** MMP-9 gene-fold change obtained by RT-PCR; **D** BCL2 gene-fold change; **E** α-SMA gene-fold change that obtained by qRT-PCR. The graphs of genes are plotted as 2^−∆∆C*t*^-fold changes. Data are expressed as mean ± SEM. A one-way ANOVA comparison test was employed to determine the significant differences (*P* < 0.05). *(*P* ≤ 0.05), **(*P* ≤ 0.01), ***(*P* ≤ 0.001), ns, (non-significant, *P* ≥ 0.05)

The study used reverse transcription polymerase chain reaction (RT-PCR) assays to figure out the effects of AgNPs and Sorafenib (Sor) on the expression levels of AMPK and mTOR. Additionally, the study investigated the influence of these substances on several markers associated with angiogenesis, metastasis, apoptosis, and autophagy, including MMP-9, BCL-2, and alpha-smooth muscle actin (α-SMA). As a comparison to the cell control, AgNPs treatment in WI-38 cells caused upregulation of AMPK and downregulation of mTOR, MMP-9, BCL-2, and α-SMA, whereas, in control cell culture, AgNPs treatment did not show a significant effect in BCL-2 compared to Sor (Fig. 9). These results revealed that AgNPs treatment had an impact on the expression of these genes, which suggests that AgNPs have the potential to regulate the expression of genes involved in cell metabolism, proliferation, and apoptosis.

**Fig. 10:**
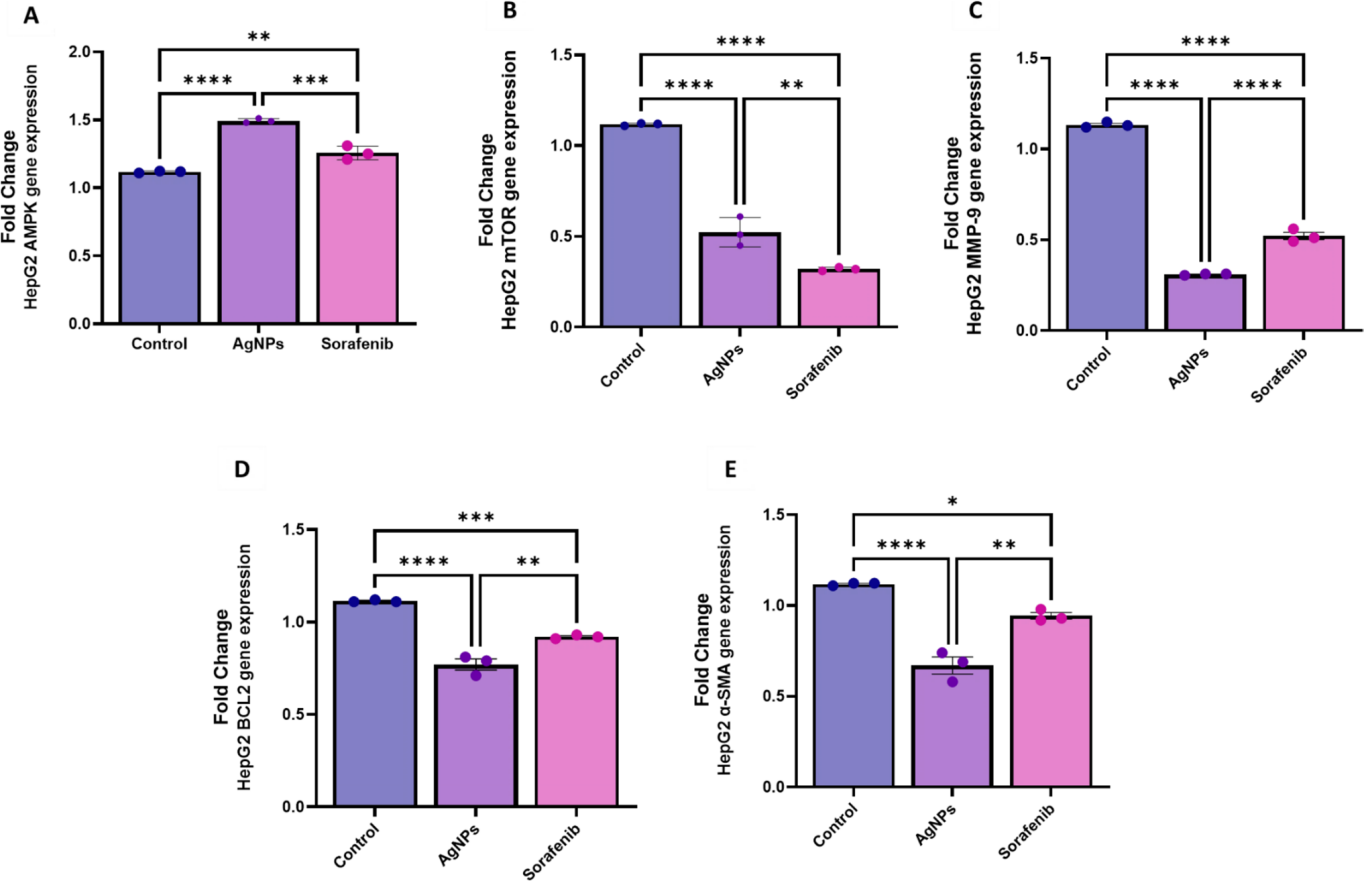
Effect of AgNPs or Sor treatment on the relative expression of apoptosis-autophagy-related genes on HepG2 cell lines **A** AMPK gene-fold change; **B** mTOR gene-fold change; **C** MMP-9 gene-fold change obtained by RT-PCR; **D** BCL2 gene-fold change; **E** α-SMA gene-fold change that obtained by qRT-PCR. The graphs of genes are plotted as 2^−∆∆C*t*^-fold changes. Data are expressed as mean ± SEM. A one-way ANOVA comparison test was employed to determine the significant differences (*P* < 0.05). *(*P* ≤ 0.05), **(*P* ≤ 0.01), ***(*P* ≤ 0.001), ns, (non-significant, *P* ≥ 0.05)

The treatment of HepG2 cells with AgNPs resulted in the most significant upregulation of AMPK compared to Sor, as revealed in (Fig. 10). A comparison of the expression of mTOR, MMP-9, BCL-2, and mTOR in HepG2 cells treated with AgNPs and the control was found to be the most strongly suppressed, compared to Sor and control. This suggests that AgNPs may have the ability to modulate the AMPK/mTOR signalling pathway, which can lead to the suppression of MMP-9, BCL-2, and α-SMA expressions, as well as an increase in apoptosis and autophagy. This modulation of the AMPK/mTOR signalling pathway by AgNPs could be responsible for the observed decrease in cell viability, decreased proliferation, and increased apoptosis and autophagy seen in HepG2 cells treated with AgNPs.

### Molecular docking and ADMET assessment

**Fig. 11:**
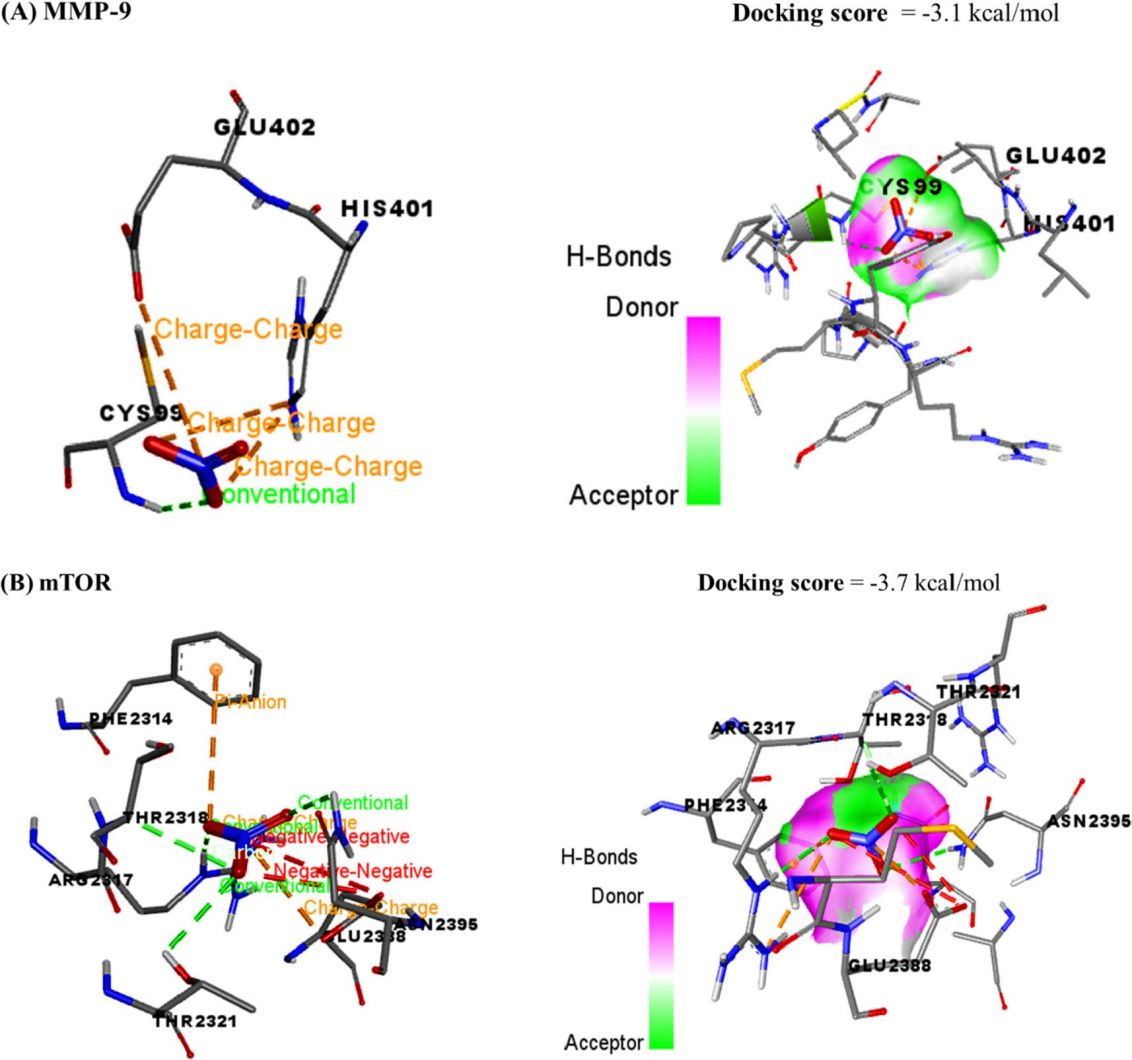
The docking interactions of AgNO3 with** A** MMP-9 and** B** mTOR. MMP-9 and mTOR binding sites were bound by the compound AgNO3, leading to binding energies of − 3.1 and − 3.7 kcal/mol, respectively

**Fig. 12:**
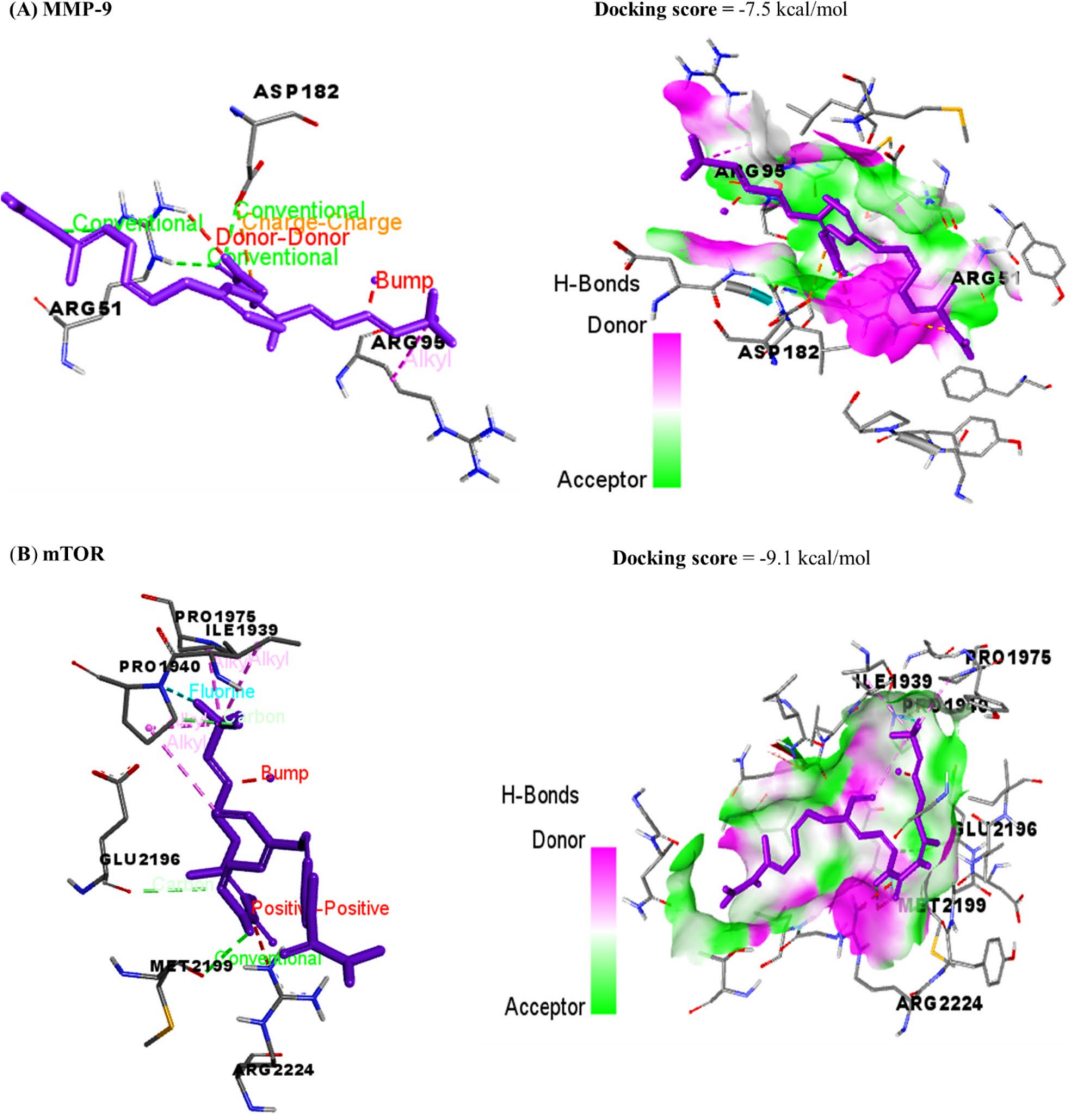
The docking interactions of sorafenib with **A** MMP-9 and** B** mTOR. The binding affinities of sorafenib to the MMP-9 and mTOR interaction sites were − 7.1 kcal/mol and − 9.5 kcal/mol, respectively

The docking interactions of AgNO3 and sorafenib with MMP-9 and mTOR are revealed in Table 1 and (Figs. 11, 12), respectively. The compound AgNO3 exhibited interactions with the binding sites of MMP-9 and mTOR, resulting in binding energies of − 3.1 and − 3.7 kcal/mol, respectively. Sorafenib had a binding affinity of − 7.5 kcal/mol and − 9.1 kcal/mol to the MMP-9 and mTOR interaction sites, respectively. The observed energies suggest a significant affinity between AgNO3 and the binding sites of MMP-9 and mTOR, as well as between sorafenib and the binding sites of MMP-9 and mTOR. This suggests that the compounds AgNO3 and sorafenib possess the capability to form complexes with MMP-9 and mTOR, hence possibly impeding their respective functionalities.

**Table 1:** Molecular interactions of AgNPs and sorafenib against matrix metalloproteinase-9 (MMP-9) and mammalian target of rapamycin (mTOR)

Drugs	Targets	Docking scores (kcal/mol)	Interaction residues	Interaction type
AgNPs	MMP-9	− 3.1	CYS99	Conventional hydrogen bond
			HIS401	Charge-charge
			GLU402	Charge-charge
	mTOR	− 3.7	PHE2314	Charge-charge
			ARG2317	Conventional hydrogen bond
			THR2318	Conventional hydrogen bond
			THR2321	Conventional hydrogen bond
			GLU2388	Charge-charge
			ASN2395	Conventional hydrogen bond
Sorafenib	MMP-9	− 7.5	ARG51	Conventional hydrogen bond
			ARG95	Hydrophobic
			ASP182	Hydrogen bond
			ASP182	Charge-charge
	mTOR	− 9.1	ILE1939	Hydrophobic
			PRO1940	Conventional hydrogen bond
			PRO1940	Halogen
			PRO1975	Hydrophobic
			GLU2196	Conventional hydrogen bond
			MET2199	Conventional hydrogen bond

The hepatotoxicity of sorafenib and the mutagenic likelihood of AgNO3 were predicted using ADMET and Ames’ methodologies, respectively. The aforementioned predictions underscore the need for further assessment of the hepatotoxicity and mutagenicity of AgNO3. The results obtained from the ADMET and Ames' predictions indicated that the hepatotoxicity of sorafenib and mutagenicity of AgNO3 exceeded initial expectations, therefore suggesting that these substances cannot be disregarded as possible dangers (Table 2). This observation underscored the need for conducting more comprehensive investigations on the hepatotoxic and mutagenic effects of AgNO3 to further our comprehension of the possible hazards it may provide.

**Table 2:** ADMET and Ames predictions of AgNO_3_ and sorafenib

	ADME prediction					Ames’ prediction
Drugs	Solubility level	BBB level	Excretion (CYP2D6)	Hepatotoxicity	PPB	
AgNO_3_	4	3	False	True	False	Mutagen
Sorafenib	1	4	False	True	True	Non-Mutagen

## Discussion

Considerable effort has been put into creating effective anti-cancer therapies in recent decades [55]. Nanomaterials of all types, including AgNPs, are considered [56]. AgNPs depend on how the targeted cell behaves biologically. Thus, they may enhance standard treatments through the induction of oxidative stress-mediated autophagy and/or apoptosis [57, 58]. In developing novel, effective therapeutic tools, the current study has focused on the possibility of using AgNPs in oncotherapy. To characterize realistic opportunities for using AgNPs to treat cancer, AgNPs have been investigated for their ability to induce apoptosis in liver cancer cells as well as their ability to play a role in the immune response to these cells. To reach this goal, we examined signalling pathways in HepG2 cells and gene expression profiles associated with apoptosis and autophagy. Furthermore, we sought to identify potential mechanisms by which these signalling pathways might be regulated.

In synthesizing AgNPs, the green biosynthesis and stabilization process by *Lactobacillus acidophilus* plays a key role. This is because it is a relatively cheap and safe process, as well as because it produces high-quality nanoparticles [59]. In synthesizing silver nanoparticles, the green biosynthesis and stabilization process by *Lactobacillus acidophilus* plays a key role. This is because it is a relatively cheap and safe process, as well as because it produces high-quality nanoparticles. Reducing aqueous silver ions to AgNPs caused the color change in the *Lactobacillus acidophilus* supernatant. The reduction process is caused by the bacteria’s ability to produce enzymes that catalyze the reduction of silver ions [12]. This process effectively produces high-quality nanoparticles because it ensures that the nanoparticles are of a consistent size and shape, which is vital for many applications [60]. Additionally, the *Lactobacillus acidophilus* supernatant helps to reduce the toxicity often associated with silver nanoparticles [61].

The FTIR method detects bond characteristics based on vibrations, identifying functional groups and revealing information regarding covalent bonds between them [62]. A significant feature of marine bacteria is that they possess functional groups, making them a potential biotechnological resource [63]. Our FTIR spectra indicate that the AgNPs exhibit these peaks, which were attributed to silanols, carboxylates, phosphonates, and siloxanes on the surface of the silver nanoparticles. These functional groups, such as carboxylates and phosphonates, are essential for bacteria to survive in the ocean environment [64]. The presence of siloxanes on the surface of the silver nanoparticles also makes them an attractive option for biotechnological applications, as these molecules can bind with other molecules, making them useful in various chemical reactions [65]. Additionally, AgNPs were more stable in a solution with a high CN-amine concentration [66]. This stability is caused by the electrostatic interactions between the AgNPs and CN-amines, which causes the AgNPs to stretch [67]. The bending of the NH side of peptides is caused by the interactions between the AgNPs and peptides, which causes the AgNPs to bend [68].

It is evident from our data that the surface plasmon resonance peak bands in the UV–Vis spectrum are centred around 425 nm, which is a characteristic feature of AgNPs. It was observed that the absorption peak at 425 nm decreased in width, and the peak intensity increased as a result. As Sankar et al. [60] suggested, the peak intensity may indicate the presence of smaller, spherical nanoparticles with some agglomeration. Our study’s results also agree with those of previously published studies [69, 70].

Our data revealed that AgNPs treatment leads the cells to apoptosis, which was visualized using AO/EB staining of the cells. This could be explained by the fact that AgNPs treatment caused a decrease in the mitochondrial membrane potential, leading to an increase in ROS, which in turn causes cells to die, a process known as apoptosis [71]. Furthermore, AgNPs treatment also induced the expression of apoptotic proteins, such as Bax and caspase-9 [72]. Additionally, Barabadi et al. [73] revealed that induced overproduction of ROS and interruption of ATP synthesis by AgNPs prevent the mitochondrial respiratory chain from functioning properly, resulting in mitochondrial pathway apoptosis. The results of their works provide strong preliminary evidence that biogenic AgNPs are effective against hepatic cancer cells in in vitro studies, which are involved in the apoptotic process [74]. These data were confirmed by the presence of the biomolecular layer on the surface of AgNPs, which is expected to play an essential role in the apoptosis induced by AgNPs in liver cancer cells [75], as described here. The present study demonstrated that 50 µg/ml AgNPs inhibited HepG2 cancer cells' growth. Significantly, HepG2 liver cancer cells responded to AgNPs by inducing intrinsic apoptotic mechanisms. As a result of interactions between AgNPs' biomolecular layer and proteins on HepG2 cancer cells, a cascade of events that lead to apoptosis may unfold. At these events, caspases, which are the main executors of apoptosis, become activated. Additionally, other proteins are activated, such as p53, which regulates apoptosis and cell cycle. There have been numerous reports describing AgNPs as an inhibitor of liver cancer cell growth both in vitro and in vivo [73, 76]. Furthermore,

In cancer treatment, the MTT assay can assess the cytotoxicity of substances by determining the number of viable cells. According to the MTT results, AgNPs were toxic to HepG2 cells at a 50 μg/ml concentration. Additionally, AgNPs showed a high ability to inhibit cell growth, with the most outstanding inhibition occurring at 100 μg/ml. This indicates that AgNPs may be a potential cancer treatment. In similar research, other researchers have documented AgNPs' cytotoxicity in several cancer cells, including oral cancer cell lines, breast cancer, and human cervical cancer [77]. This could be attributed to AgNPs causing oxidative stress and membrane damage in cells, which can lead to cell death. Furthermore, AgNPs can cause apoptosis, a programmed cell death process, in cancer cells and could potentially kill cancer cells, making them a potential cancer treatment [78].

The AgNPs treatment significantly augmented the concentration of GSH in these cells, neutralizing both MDA and GSH levels. A single Sor was added to both HepG2 and WI-38 cells. However, when HepG2 and WI-38 cells were coated with AgNPs, MDA levels decreased significantly, while those treated with Sor increased. This suggests that AgNPs have an antioxidant effect, while Sor has an antioxidant and pro-oxidant effect. The antioxidant effect of AgNPs is likely due to its ability to bind to free radicals and reduce their activity, while the pro-oxidant effect of Sor is likely due to its ability to oxidize and damage proteins, lipids, and nucleic acids [79].

A further observation demonstrating AgNPs’ apoptosis-inducing ability was IL-33 and TNF-α secretion by cells treated with AgNPs. Cancerous cells have been reported to secrete pro-inflammatory cytokines, which regulate host immune responses [57]. IL-33 and TNF-α are pivotal in regulating inflammation and immune responses [80]. The release of these cytokines by AgNPs is thought to cause the observed apoptosis-inducing ability [81]. This suggests that the AgNPs may be able to activate the apoptosis pathway in cancer cells and induce the activation of other immune cells, such as dendritic cells and natural killer cells, which may lead to a more effective immune response [82, 83]. It is worth noting that the cytokines IL-33 and TNF-α, which were observed in the supernatants of the cells treated with AgNPs, have previously been documented as being produced by cancer cells undergoing apoptosis and playing a role in the signalling of dendritic and natural killer cells [84, 85].

One of the well-known characteristics of cancer cells is their ability to avoid apoptosis. Furthermore, autophagy suppresses tumorigenesis in cancer cells by inhibiting their survival and inducing their death [86]. Therefore, we analyzed intrinsic and extrinsic genes associated with apoptosis and autophagy in our study. AgNPs significantly upregulated AMPK expression, while AgNPs significantly suppressed mTOR, MMP-9, BCL-2 and α-SMA expression. Our results provide evidence that AgNPs have the potential to regulate apoptosis and autophagy in cancer cells, which could be a promising therapeutic approach for cancer treatment. As mTOR/BCL-2 regulates the apoptotic pathway, this study may be supported by its classification as an anti-apoptotic protein. The results here reveal that AgNPs cause the HepG2 cell line to enter apoptosis by downregulating BCL-2. Several studies have linked AgNPs to apoptosis by lowering mTOR/BCL-2 in cancerous cells [87, 88]. However, apoptosis can be sustained depending on the balance between cell division and cell death.

Novel targeted therapies capable of inducing apoptosis or enhancing cancer cells’ susceptibility to established cytotoxic medicines have been successfully created. This can be attributed to the increasing ubiquity of intrinsic and extrinsic apoptotic triggering mechanisms. AgNPs induce apoptosis, inhibiting the AMPK/mTOR pathway [37]. This study indicates that the AMPK signalling pathway was effectively repressed in HepG2 cells. In contrast, the translocation of mTOR and BCL-2 was notably diminished in cells treated with AgNPs. These results imply that AMPK inhibition may be implicated in apoptosis. The observed behaviour can be attributed to AMPK's crucial role as a metabolic sensor and its involvement in regulating cell growth. Furthermore, AMPK negatively regulates the mTOR signalling pathway, inhibiting cancer proliferation and growth [88]. Integrating signals from the PI3K/Akt pathway, AMPK also governs cell survival, proliferation, and angiogenesis [89].

Moreover, it is worth noting that HCC commonly exhibits elevated mTOR levels associated with early recurrence and a poor prognosis [25, 90]. Consequently, targeting mTOR inactivation has been proposed as a potential therapeutic approach to limiting cancer cell growth in HCC. Thus, the obtained findings align with our theoretical framework, suggesting that AgNPs trigger autophagy via AMPK/mTOR/BCL-2 signalling pathway and that autophagy plays a pivotal role in AgNPs-induced cellular collapse.

Molecular docking analysis has confirmed the antiproliferative and apoptotic effects of AgNPs on HepG2 cells. This study has found the interaction between AgNO3 and Sor and their potential targets, including MMP-9 and mTOR. The genes under investigation have considerable significance in converging apoptotic and proliferation pathways. The computational analysis results demonstrated that using AgNPs may efficiently suppress the activity of MMP-9 and mTOR. The observed suppression led to a decrease in the expression of MMP-9, which ultimately resulted in a reduction of the expression of mTOR. The inhibition of the MMP-9-BCL-2-dependent AMPK activation pathway by AgNPs leads to the suppression of mTOR activation, hindering the subsequent pathways linked to cancer proliferation.

Moreover, via the mechanism of AMPK downregulation, AgNPs significantly diminish the intracellular levels of AMPK, decreasing the abundance of mTOR available to initiate downstream signalling cascades. This could be explained by the fact that AMPK is a crucial regulator of mTOR, and its downregulation by AgNPs leads to a decrease in the activity of mTOR, which would otherwise initiate a cascade of signalling events that would lead to the activation of the pathways associated with cell proliferation and differentiation [91]. For instance, the AMPK/mTOR pathway is essential for the translation of proteins involved in cell growth and proliferation, and a decrease in the abundance of AMPK can thus inhibit cell growth [92].

## Conclusion

This study shows that AgNPs exhibit an inhibitory effect on cell proliferation and induce apoptosis in hepatocellular carcinoma HepG2 cells. The AMPK/mTOR inhibitory signalling pathway plays a substantial role in developing HCC. The present study elucidates the potential apoptotic anti-HCC characteristics of AgNPs by suppressing the AMPK/mTOR signalling pathway. The cytotoxic action of AgNPs was observed to hinder the proliferation of HepG2 cells, with an IC_50_ of 5.21 µg/ml. In the research on apoptotic activity, it was observed that the administration of AgNPs resulted in a considerable induction of apoptotic cell death in HCC cells. This effect was observed following a 48-h treatment with AgNPs, which led to the release of pro-inflammatory cytokines. In addition, the highest levels of mTOR, MMP-9, BCL-2, and α-SMA inhibition coincide with the upregulation of AMPK expression in HepG2 cells treated with AgNPs. This study investigated the AMPK/mTOR signalling pathway of AgNPs through integrated methodologies both in vitro and in silico. The cumulative findings of our research provide more evidence in favour of the potential anti-cancer efficacy of AgNPs as a viable alternative therapy strategy for HCC. Nevertheless, it is important to carry out further in vivo research using animal models to authenticate these results. AgNPs can selectively target tumor cells owing to their distinct structure and composition. Moreover, they can selectively attach to certain receptors located on the neoplastic cells, augmenting their efficacy in inducing cell death. Additionally, the AgNPs exhibit immunostimulatory properties, facilitating further suppression of tumor cells.

### Supplementary Information

Below is the link to the electronic supplementary material.

Supplementary file1 (DOCX 16 KB)

## Data Availability

The datasets generated and/or analyzed during the current study are available in the GeneBank repository with accession number OR616671.
