# Supplementary material for: Probiotic-derived silver nanoparticles target mTOR/MMP-9/BCL-2/dependent AMPK activation for hepatic cancer treatment
Source: Med Oncol. 2024 Apr 4;41(5):106. doi: 10.1007/s12032-024-02330-8 (PMC10995097; doi:10.1007/s12032-024-02330-8)
Supplement: Supplementary file 1 — Supplementary file1 (DOCX 16 KB) [file 12032_2024_2330_MOESM1_ESM.docx]

**Table S1:**  RT-PCR primer sequence for expression analysis

| Gene | Forward primer | Reverse primer |
| --- | --- | --- |
| AMPK | 5’ ACCAGGTCATCAGTACACCA 3’ | 5’ ATTGTGGCCCTCTTCATGGG 3’ |
| mTOR | 5’ GCCGCGCGAATATTAAAGGA 3’ | 5’ CTGGTTTCCTCATTCCGGCT 3’ |
| MMP-9 | 5′ ACGACGTCTTCCAGTACCGA 3′ | 5′ TTGGTCCACCTGGTTCAACT 3′ |
| BCL2 | 5’ AGGAAGTGAACATTTCGGTGAC 3’ | 5’ GCTCAGTTCCAGGACCAGGC 3’ |
| α-SMA | 5′ GAAGAAGAGGACAGCACTG 3′ | 5′ TCCCATTCCCACCATCAC 3′ |
| GAPDH | 5’ TGCACCACCAACTGCTTAGC 3’ | 5’ GGCATGGACTGTGGTCATGAG 3’ |
